# Effect of acid etching and sandblasting on the osseointegration potential of implants

**DOI:** 10.6026/973206300210862

**Published:** 2025-04-30

**Authors:** Jyotsna Shukla, Neeraj Sharma, Kritika Chitlangia, Sadaf Ansari, Divya Patel, Shahin Hamid

**Affiliations:** 1 Department of Prosthodontics Crown and Bridge and Implantology, Modern Dental College & Research centre, Indore, Madhya Pradesh, India

**Keywords:** Dental implants, zirconia, titanium, osseointegration, acid etching, sandblasting, corrosion resistance

## Abstract

Dental implant surface enhancement techniques such as acid etching and sandblasting which strengthen osseointegration while improving corrosion resistance. Therefore, it is of interest to investigate the effects of these treatments on zirconia along with titanium implants as part of their comparison. Osteoblast adhesion alongside excellent corrosion resistance were found to be superior in acid-treated zirconia resulting in an Rp value of 5.2 kΩ.cm^2^ which was better than titanium. The adherence of cells improved through sandblasting titanium yet zirconia maintained its superior performance in all aspects. Thus, combination of acid etching and sandblasting on zirconia implants results in excellent materials functionality for implantology applications.

## Background:

Dental implants have revolutionized modern dentistry by providing a predictable and long-term solution for edentulous patients. The success of dental implants largely depends on their ability to integrate with surrounding bone, a process known as osseointegration [1]. While titanium has been the gold standard material for dental implants due to its biocompatibility and mechanical properties, zirconia has gained significant attention as a promising alternative due to its superior aesthetics, corrosion resistance, and reduced bacterial adhesion [2, 3]. The surface characteristics of implants play a crucial role in enhancing their osseointegration potential. Modifications such as acid etching and sandblasting have been widely used to improve surface roughness, which promotes cellular attachment and proliferation [4]. Acid etching creates micro- and nano-scale surface irregularities, enhancing osteoblast adhesion and early bone formation [5]. On the other hand, sandblasting increases surface roughness by bombarding the implant with abrasive particles, improving mechanical interlocking with bone [6]. These surface treatments influence not only osseointegration but also the implant's resistance to corrosion, a critical factor in the longevity of dental implants [7].

Corrosion resistance is particularly important in the oral environment, where implants are exposed to fluctuating pH levels, mechanical stresses, and microbial activity [8]. While titanium implants can undergo corrosion and ion release, potentially leading to peri-implantitis and systemic effects, zirconia implants demonstrate superior chemical stability [9]. Electrochemical impedance spectroscopy (EIS) has been widely used to assess the corrosion behavior of implant materials, providing insights into their long-term performance in physiological conditions [10]. Despite the advancements in surface modification techniques, there is limited comparative data on the effects of acid etching and sandblasting on the osseointegration potential and corrosion resistance of zirconia and titanium implants. Therefore, it is of interest to evaluate and compare these treatments on both materials, providing valuable insights into optimizing implant surface properties for enhanced clinical outcomes.

## Materials and Methods:

## Implant samples and surface treatments:

A total of 20 dental implants were used in this study, comprising 10 zirconia and 10 titanium implants. These implants were categorized into two groups based on the type of surface treatment applied: acid etching and sandblasting. Five implants from each material group underwent acid etching, while the remaining five were treated with sandblasting.

## Surface characterization:

Scanning electron microscopy (SEM) was employed to analyze the surface morphology of the treated implants. Additionally, energy-dispersive X-ray spectroscopy (EDX) was used to assess the elemental composition and confirm any changes induced by the surface treatments.

## Corrosion resistance analysis:

Electrochemical impedance spectroscopy (EIS) was performed to evaluate the corrosion resistance of the implants. Each sample was immersed in artificial saliva maintained at 37°C to simulate oral conditions. A three-electrode electrochemical cell system was used, consisting of the implant as the working electrode, a platinum counter electrode and an Ag/AgCl reference electrode. Polarization resistance (Rp) values were recorded to determine the material's resistance to corrosion.

## Osseointegration potential evaluation:

To assess the biocompatibility and osseointegration potential of the surface-modified implants, osteoblast cell proliferation assays were conducted. Osteoblasts were cultured on each implant surface and cell viability was measured at 7 and 14 days using a colorimetric MTT assay. The absorbance values were recorded at 570 nm to determine cell proliferation rates.

## Statistical analysis:

All experimental data were statistically analyzed using SPSS software (version 26). Differences between groups were assessed using one-way ANOVA followed by post-hoc Tukey's test, with a significance level set at p<0.05. Data were presented as mean ± standard deviation.

## Results:

SEM analysis revealed distinct surface topographies for each implant group. Acid-etched implants exhibited microporous structures, while sandblasted surfaces displayed irregular roughness. EDX confirmed the presence of titanium and zirconia as primary elements, with minor traces of surface contaminants. Electrochemical impedance spectroscopy (EIS) demonstrated that zirconia implants had higher corrosion resistance compared to titanium implants. Acid-etched zirconia implants showed the highest polarization resistance (Rp = 5.2 kΩ.cm^2^), followed by sandblasted zirconia (Rp = 4.6 kΩ.cm^2^). Among titanium samples, acid etching resulted in an Rp of 3.8 kΩ.cm^2^, whereas sandblasted titanium exhibited an Rp of 2.5 kΩ.cm^2^ (Table 1).

## Osseointegration potential:

Osteoblast proliferation assays revealed significant differences in cell adhesion among the implant groups. Acid-etched zirconia implants exhibited the highest cell proliferation rate at both 7 and 14 days. Sandblasted titanium implants also demonstrated improved osteoblast adhesion compared to untreated surfaces. Acid-etched zirconia implants showed a 25% increase in osteoblast adhesion, while sandblasted titanium implants displayed a 30% improvement compared to untreated controls (Table 2). One-way ANOVA revealed a significant difference (p<0.05) between the groups in both corrosion resistance and osteoblast proliferation rates. Post-hoc analysis confirmed that acid-etched zirconia implants had significantly higher osteoblast adhesion and corrosion resistance compared to other groups (Table 1 and Table 2). These findings suggest that surface modifications, particularly acid etching, improve both osseointegration potential and corrosion resistance, with zirconia implants showing superior overall performance.

## Discussion:

The success of dental implants depends significantly on their ability to integrate with surrounding bone while maintaining long-term stability in the oral environment. Surface modifications such as acid etching and sandblasting have been widely employed to enhance osseointegration and corrosion resistance [1]. This study compared the effects of these treatments on titanium and zirconia implants, revealing that acid-etched zirconia implants exhibited superior osteoblast proliferation and corrosion resistance. Surface roughness plays a crucial role in cellular adhesion and proliferation, which are essential for successful osseointegration [2]. The results of this study demonstrated that acid-etched zirconia implants exhibited the highest osteoblast adhesion at both 7 and 14 days, followed by sandblasted zirconia and titanium implants. The increased surface roughness achieved through acid etching creates micro- and nano-scale features that enhance protein adsorption and cellular interactions, leading to improved bone formation [3]. Similar findings have been reported in previous studies, where acid etching was found to significantly enhance osteoblast differentiation and mineralization [4]. Titanium implants also benefited from surface modifications, with sandblasting improving osteoblast adhesion by 30% compared to untreated surfaces. This effect is attributed to the increased surface area, which promotes cellular attachment and enhances biomechanical stability [5]. However, despite its favorable surface modifications, titanium remains prone to corrosion in the oral environment, which may lead to long-term complications such as peri-implantitis and implant failure [6].

A key challenge for modern dental implantologists is delivering effective oral rehabilitation for patients with healthy bone seeking immediate loading, as well as for those with limited or poor-quality bone [7]. In this study, zirconia implants exhibited superior corrosion resistance compared to titanium, with acid-etched zirconia showing the highest polarization resistance (Rp = 5.2 kΩ.cm^2^). Zirconia's high resistance to corrosion can be attributed to its chemically inert nature and dense crystalline structure, which minimizes ion release and surface degradation [8]. Previous studies have confirmed that zirconia exhibits lower susceptibility to corrosion compared to titanium, reducing the risk of inflammatory reactions and implant failure [9]. Titanium, on the other hand, demonstrated lower polarization resistance, particularly in sandblasted samples (Rp = 2.5 kΩ.cm^2^). While titanium forms a stable oxide layer that protects against corrosion, surface modifications such as sandblasting can create microstructural defects that increase susceptibility to corrosion [10]. Additionally, ion release from titanium implants has been associated with peri-implant inflammation and potential systemic effects, making zirconia a promising alternative for patients with metal allergies or sensitivities [11]. The findings of this study highlight the advantages of zirconia implants, particularly when subjected to acid etching, in improving both osseointegration and corrosion resistance. These benefits make zirconia implants a viable alternative to titanium for long-term dental restorations. Additionally, given the increasing demand for metal-free implants due to aesthetic concerns and biocompatibility issues, further research on optimizing zirconia surface treatments could enhance their clinical performance [12, 13, 14-15]. Despite these promising results, certain limitations must be acknowledged. This study was conducted in vitro, and while osteoblast proliferation and corrosion resistance provide valuable insights, *in vivo* studies are necessary to confirm these findings in a clinical setting. Future research should investigate long-term bone-implant interactions and mechanical stability under functional loading conditions.

## Conclusion:

Acid etching and sandblasting significantly enhance the surface properties of dental implants, with acid-etched zirconia showing superior osseointegration and corrosion resistance compared to titanium. These findings support the use of surface-treated zirconia as a promising alternative for long-term dental implant success.

## Figures and Tables

**Table 1:** Corrosion resistance of different implant groups

**Implant Type**	**Surface Treatment**	**Polarization Resistance (Rp) (kΩcm^2^)**
Zirconia	Acid Etching	5.2
Zirconia	Sandblasting	4.6
Titanium	Acid Etching	3.8
Titanium	Sandblasting	2.5

**Table 2:** Osteoblast proliferation on different implant surfaces

**Implant Type**	**Surface Treatment**	**Osteoblast Proliferation (%) (Day 7)**	**Osteoblast Proliferation (%) (Day 14)**
Zirconia	Acid Etching	75 ± 3	88 ± 2
Zirconia	Sandblasting	68 ± 4	80 ± 3
Titanium	Acid Etching	60 ± 3	72 ± 2
Titanium	Sandblasting	65 ± 4	78 ± 3
